# The impact of age and frailty on hospitalization and survival in older liver transplant recipients: a longitudinal cohort study

**DOI:** 10.3389/fragi.2025.1539688

**Published:** 2025-04-28

**Authors:** Matteo Valenti, Chiara Ceolin, Marco Rossato, Chiara Curreri, Maria Devita, Marta Tonon, Carlotta Campodall’Orto, Jessica Vanin, Martina Gambato, Umberto Cillo, Patrizia Burra, Paolo Angeli, Giuseppe Sergi, Marina De Rui

**Affiliations:** ^1^ Department of Medicine - DIMED, University of Padua, Italy; ^2^ Division of Geriatrics, DIDAS Medicine Department, University - Hospital of Padua, Padua, Italy; ^3^ Department of Neurobiology, Care Sciences and Society, Karolinska Institutet and Stockholm University, Aging Research Center, Stockholm, Sweden; ^4^ Department of General Psychology - DPG, University of Padua, Italy; ^5^ Division of Hepatobiliary Surgery and Liver Transplantation, DIDAS Surgery Department, University - Hospital of Padua, Padua, Italy; ^6^ Multivisceral Transplant Unit, DIDAS Medicine Department, University - Hospital of Padua, Padua, Italy, University of Padova, Padua, Italy; ^7^ Department of Surgery, Oncology and Gastroenterology - DISCOG, University of Padua, Italy; ^8^ Gastroenterological Endoscopy Unit, DIDAS Medicine Department, University - Hospital of Padua, Padua, Italy

**Keywords:** older adults, liver transplantation, MELD, frailty, CTP

## Abstract

**Purpose:**

Frailty is a well-established risk factor for adverse outcomes, particularly in liver transplant candidates. This study investigates the impact of age and frailty on key clinical outcomes—hospitalizations, waitlist survival, and post-transplant mortality—in cirrhotic patients evaluated for liver transplantation.

**Methods:**

This study included older adults with chronic liver disease under consideration for transplantation. Data collected encompassed medical history, Model for End-Stage Liver Disease (MELD) and Child-Turcotte-Pugh (CTP) scores, Mini-Mental State Examination (MMSE), Mini Nutritional Assessment (MNA), and frailty status, assessed using both the Liver Frailty Index (LFI) and the Survey of Health, Ageing, and Retirement in Europe Frailty Index (SHARE-FI). Clinical outcomes, including mortality and hospitalizations, were tracked over a 24-month period.

**Results:**

Among 100 patients (67% male), those under 70 exhibited higher MNA, MMSE, and SHARE-FI scores. Based on frailty classification, 25 patients were frail, 28 pre-frail, and 47 robust. Younger patients experienced more hospitalizations during follow-up (p = 0.03) and had a higher probability of hospitalization within 24 months (p = 0.002). Although transplant-free survival did not differ significantly across groups, frail patients had a significantly higher mortality rate (p = 0.04). Overall, 24 patients underwent transplantation, while 26 died, including six post-transplant deaths. MELD and CTP scores were strong predictors of mortality, while among frailty measures, only SHARE-FI demonstrated significant predictive value. In multivariate Cox models, MELD [HR = 1.17, p = 0.001; HR = 1.11, p = 0.002], CTP [HR = 1.43, p = 0.003; HR = 1.41, p = 0.006], and LFI (HR = 1.69, p = 0.04) were significantly associated with mortality.

**Conclusion:**

Frailty, rather than age, emerges as a key predictor of mortality in liver transplant candidates. Further research is needed to validate these findings and enhance frailty assessment, ultimately improving candidate selection for transplantation.

## Introduction

Liver disease is a major global health concern, affecting approximately 800 million people annually and contributing to nearly 2 million deaths each year (Marcellin and Kutala, 2018). The primary causes include viral infections, excessive alcohol consumption, and non-alcoholic fatty liver disease (NAFLD), while less common etiologies involve autoimmune disorders (e.g., primary sclerosing cholangitis, primary biliary cirrhosis, autoimmune hepatitis) and genetic conditions (e.g., hemochromatosis, α1-antitrypsin deficiency, Wilson’s disease) (Marcellin and Kutala, 2018; Huang et al., 2023). Without appropriate treatment, these conditions can progress to cirrhosis, which initially manifests in a compensated phase before advancing to decompensated cirrhosis, characterized by severe complications and, ultimately, end-stage liver disease (D’Amico et al., 2006). The European Association for the Study of the Liver (EASL) recommends liver transplantation for patients with end-stage disease when it is expected to improve survival or quality of life (European Association for the Study of the Liver, 2016).

With advancements in surgical techniques, liver transplantation criteria have broadened, and age is no longer considered a strict limitation for inclusion on the transplant list (Su et al., 2016). While the benefits of restoring liver function through transplantation are well established, the impact of frailty on pre-transplant evaluation and outcomes remains a subject of ongoing debate (Chen et al., 2014). Frailty is a clinical geriatric syndrome characterized by age-related declines in physiological reserves and an increased vulnerability to stressors. Its prevalence rises with age, affecting more than 25% of individuals aged 85 and older (Fried et al., 2021). In chronic liver disease, frailty arises not only as a consequence of aging but also as a direct result of the systemic alterations induced by the disease—including progressive loss of physiological reserves, sarcopenia, malnutrition, and cognitive decline. These factors, compounded by comorbidities, often impair a patient’s ability to withstand major surgery and recover from postoperative complications, even in relatively young individuals with cirrhosis. Consequently, assessing frailty in liver transplant candidates is essential because it provides a comprehensive evaluation of overall health beyond liver function alone. Frailty is known to be associated with adverse outcomes, including falls, disability, hospitalization, and mortality (Hurria et al., 2014). In the context of liver transplantation, it has been linked to higher in-hospital mortality, increased perioperative complications, and greater post-transplant healthcare costs (Lai et al., 2022; Porter et al., 2024). Notably, previous studies have demonstrated that frailty—independent of age—doubles the risk of mortality for patients on the transplant waitlist (Haugen et al., 2020), underscoring its critical role in waitlist outcomes across all age groups. Furthermore, pre-transplant frailty is a strong predictor of post-transplant prognosis, with frail patients exhibiting higher post-transplant mortality, prolonged hospital stays, and increased rates of non-home discharge (Lai et al., 2022). In light of this evidence, the American Society of Transplantation advocates for the integration of frailty assessment into standard pre-transplant evaluations to provide a more comprehensive assessment of transplant candidates (Romero-Ortuno, 2013). However, the current literature on the impact of frailty in older patients undergoing or being considered for liver transplantation remains highly heterogeneous, largely due to the inconsistent use of frailty assessment tools (Vogliotti et al., 2023).

Building on existing evidence, this study aims to investigate the effects of both age and frailty on clinical outcomes and mortality among old patients evaluated for liver transplantation. Specifically, we will examine the onset of decompensation symptoms, hospitalization rates, listing and delisting processes, waitlist survival, and post-transplant mortality in cirrhotic patients. This research seeks to provide a comprehensive understanding of how frailty, as a measure of overall physiological reserve, interacts with age to influence outcomes, ultimately informing improved patient selection and management strategies in liver transplantation.

## Patients and methods

### Study population

This observational study was conducted on patients attending the outpatient clinics of the Regional Center for Liver Diseases within the Clinical Medicine 5 Department, in collaboration with the Multivisceral Transplant and Hepatobiliary Surgery Units of the University Hospital of Padua. The study was carried out by a highly specialized team in liver diseases and transplantation. As part of this multidisciplinary approach, our comprehensive geriatric assessment was integrated into the care pathway established by internists and surgeons. All patients, recruited between December 2018 and November 2022, were followed for at least 2 years, during which data on disease complications, hospitalizations, and mortality were collected. Additionally, all included patients were aged ≥65 years. The inclusion criteria were as follows: diagnosis of advanced liver cirrhosis and listing for liver transplantation; ability to provide informed consent for the use of data in clinical research; capability to undergo physical performance tests.

The study protocol adhered to good clinical practice guidelines and the ethical standards outlined in the 1964 Declaration of Helsinki, revised in 2000, and was approved by the local Ethics Committee (Comitato Etico per la Sperimentazione Clinica della Provincia di Padova, protocol number 0014675). Participants received a comprehensive explanation of the potential risks and benefits of participation and provided both oral and written informed consent for data publication.

### Data collected

The following information was collected from each participant by trained physicians.

#### Patients’s characteristics

Physiological, clinical, and pharmacological data were collected from each participant during a medical interview with a skilled physician. Smoking and alcohol consumption habits, social and environmental conditions, and Model for End stage Liver Disease (MELD), and Child-Turcotte-Pugh score (CTP) scores were reported. Moreover, information related to liver disease such as etiology, prognostic scores, presence of clinical signs of decompensation (including refractory ascites, spontaneous bacterial peritonitis, overt episodes of hepatic encephalopathy, gastrointestinal bleeding, and development of hepatorenal syndrome) any related hospitalizations until the time of transplantation or death, and recent hospitalizations, were collected. Dates of listing for transplantation and removal from the list, as well as dates of transplantation or death, were also recorded.

#### Multidimensional evaluation

The geriatric multidimensional assessment has been previously described (Vogliotti et al., 2023). In summary, it included evaluating comorbidities using the Cumulative Illness Rating Scale (CIRS), nutritional status with the Mini Nutritional Assessment (MNA), functional status through Activities of Daily Living (ADL) and Instrumental Activities of Daily Living (IADL), and cognitive status with the Mini-Mental State Examination (MMSE). Body weight and height were measured with participants wearing light indoor clothing and no shoes, and the body mass index (BMI) was calculated as weight divided by height squared (kg/m^2^).

#### Frailty assessment

To assess frailty, we calculated the Liver Frailty Index [LFI- (Lai et al., 2019)] and the Survey of Health, Ageing and Retirement in Europe Frailty Index [SHARE-FI- (Romero-Ortuno, 2013)] using dedicated tools. In the Liver Frailty Index, patients were categorized into three groups: robust with a score below 3.2, pre-frail with a score between 3.2 and 4.4, and frail with a score of 4.5 or higher. Regarding the SHARE-FI, frailty was evaluated through a specific algorithm, differentiated by gender. For women: non-frail with a score below 0.32, pre-frail with a score between 0.32 and 2.13, and frail with scores above 2.13; for men: non-frail with a score below 1.21, pre-frail with a score between 1.21 and 3.01, and frail with scores above 3.01.

### Statistical analysis

For the study purposes, patients were divided into two groups based on their age at baseline (the first group comprised patients aged 70 years or younger, while the second group included patients older than 70 years) and according to SHARE-FI scores (robust, pre-frail and frail groups). The characteristics of the sample compared by age groups and frailty status are expressed as means ± standard deviation for the continuous quantitative variables with a normal distribution, and as medians (interquartile range-IQR) for those with a non-normal distribution. The normality of the distributions of the continuous quantitative variables was verified by the Shapiro–Wilk test. Categorical variables are expressed in numbers (percentages). The characteristics of the study participants were compared according to their degree of frailty using the Student’s t-test or Chi-square test depending on the type of variable. We conducted Kaplan-Meier analyses to evaluate 24-month survival and hospitalization risk, stratifying the results by age groups and SHARE-FI scores. Additionally, with a sensivity analyses, Kaplan-Meier curves were performed both in patients with only Hepatocellular Carcinoma (HCC) and considering patients MMSE scores, categorized as low or high according to the median value. The abilities of MELD, CTP, SHARE-FI and LFI to predict mortality were compared by receiver operating characteristic (ROC) curve analysis, and measurement of the area under the curve (AUC). We analyzed the predictors of overall survival in the entire sample using Cox regression, focusing on MELD, CTP, and frailty indicators. Five models were tested: Model 1 was adjusted for sex, age, and liver disease etiology; Model 2 included these variables along with MELD and SHARE-FI; Model 3 was adjusted for MELD and LFI. Model 4 accounted for sex, age, liver disease etiology, CTP, and SHARE-FI, while Model 5 included CTP and LFI as additional predictors. A sensitivity analysis was performed only in patients with HCC. The statistical tests were considered significant at p < 0.05. All analyses were performed with IBM SPSS Statistics 29.0 (Armonk, NY: IBM Corp).

## Results

### Characteristics of the sample

Table 1 presents the baseline characteristics of the sample, stratified by age groups. Among the total participants, 40 were aged 70 years or younger, while 60 were over 70 years old. The proportion of males was comparable between the two groups. Regarding etiology, 23% of patients had HCV, 14% had HBV, 31% had alcohol-related liver disease, and 15% had NASH. Alcohol-related liver disease was significantly more prevalent in the younger group compared to the older group (44.5% vs. 21.6%, p = 0.002). Conversely, HCC was more frequently observed in older patients, whereas ascites was more common among younger individuals. In the multidimensional assessment, the CIRS comorbidity index and functional scores did not differ significantly between the two groups (p = 0.34 and p = 0.55, respectively). However, MNA and MMSE scores were significantly lower in the younger group compared to the older group [MNA: 22.0 (20.0–23.6) vs 24.5 (21.0–26.5), p = 0.002; MMSE: 26.2 ± 3.4 vs 28.1 ± 1.5, p = 0.01]. Additionally, the SHARE-FI score was significantly higher in the younger group.

**TABLE 1:** Characteristics of the sample at baseline, all sample and by age groups.

Variable	All (n = 100)	Age ≤70 (n = 40)	Age>70 (n = 60)	p-value
Male sex	67 (67%)	27 (67.5%)	39 (65%)	0.14
Weight (kg)	76.7 ± 14.4	83.4 ± 17.1	72.5 ± 10.6	**0.001**
BMI (kg/m^2^)	25.5 ± 4.5	24.8 ± 3.4	26.3 ± 5.9	0.21
Etiology				
HCV	23 (23%)	9 (22.5%)	14 (23.3%)	0.65
HBV	14 (14%)	4 (11.2%)	10 (16.6%)	0.78
Alcool	31 (31%)	18 (44.5%)	13 (21.6%)	0.002
NASH	15 (15%)	4 (11.2%)	11 (18.3%)	0.24
Altro	17 (17%)	5 (12.5%)	12 (20.0%)	0.30
HCC	54 (54%)	13 (32.5%)	41 (68.3%)	**0.001**
MELD at baseline	14 (10–19)	14 (11–17)	13 (9–19)	0.78
CTP at baseline	7 (6–10)	8 (6.5–10)	7 (6–9.3)	0.14
Hospitalizations in the preceding 3 months	41 (41%)	14 (35%)	27 (45%)	0.35
Ascites	46 (46%)	24 (60%)	22 (36.6%)	**0.02**
Esophageal varices	55 (55%)	25 (62.5%)	30 (50%)	0.45
Hepatic encephalopathy	24 (24%)	12 (30%)	12 (20%)	0.24
Multidimensional evaluation				
CIRS-CI	2 (2; 4)	2 (1; 3)	3 (2; 4)	0.34
ADL	6 (5; 6)	6 (6; 8)	6 (5; 6)	0.55
IADL	7 (6; 8)	6 (5; 6)	7 (6; 8)	0.76
MNA	23.0 (21.0; 25.5)	22.0 (20.0; 23.6)	24.5 (21.0; 26.5)	**0.002**
MMSE	26.7 ± 3.2	26.2 ± 3.4	28.1 ± 1.5	**0.01**
SHARE-FI	1.45 (−0.71; 2.82)	2.25 (−0.29; 4.15)	0.82 (−0.74; 2.53)	**0.028**
Liver Frailty Index	4.04 (3.69; 4.57)	4.14 (3.69; 5.21)	3.96 (3.69; 4.32)	0.21

Notes: Numbers are expressed as number (percentages), median (IQR) or mean ± standard deviation. Significant p-values are reported in bold.

Abbreviations: HCV, Hepatitis C Virus; HBV, Hepatitis B Virus; NASH, Non-Alcoholic SteatoHepatitis; HCC, hepatocellular carcinoma; MELD, model for end stage liver disease; CTP, Child-Turcotte-Pugh score; BMI, body mass index; ADL, activity of daily living; IADL, instrumental activity of daily living; MNA, mini nutritional assessment; MPI, multidimensional prognostic index; SPMSQ, short portable mental status questionnaire; MMSE, mini mental state examination; SHARE-FI, frailty instrument of the survey of health, Ageing and Retirement in Europe.

Frailty assessment identified 25 frail patients, 28 pre-frail patients, and 47 robust patients. Frail individuals were significantly younger than the other two groups [67.0 (56.5–71.0) vs 71.0 (60.3–72.0) vs 72.0 (69.3–73.0), p = 0.011]. They also exhibited more severe liver disease, with higher MELD scores [21 (14–25) vs 18 (12–21) vs 12 (8–15), p = 0.003], but a lower prevalence of HCC. Clinically, frail patients had higher rates of ascites (72% vs. 50.0% vs 29.9%, p = 0.002), esophageal varices, and hepatic encephalopathy. Furthermore, they demonstrated lower scores in MNA, ADL, and IADL (p < 0.001 for all). (Refer to Supplementary Table S1 for additional details.).

### Evaluation of clinical outcomes by age group before liver transplantation

**FIGURE 1:**
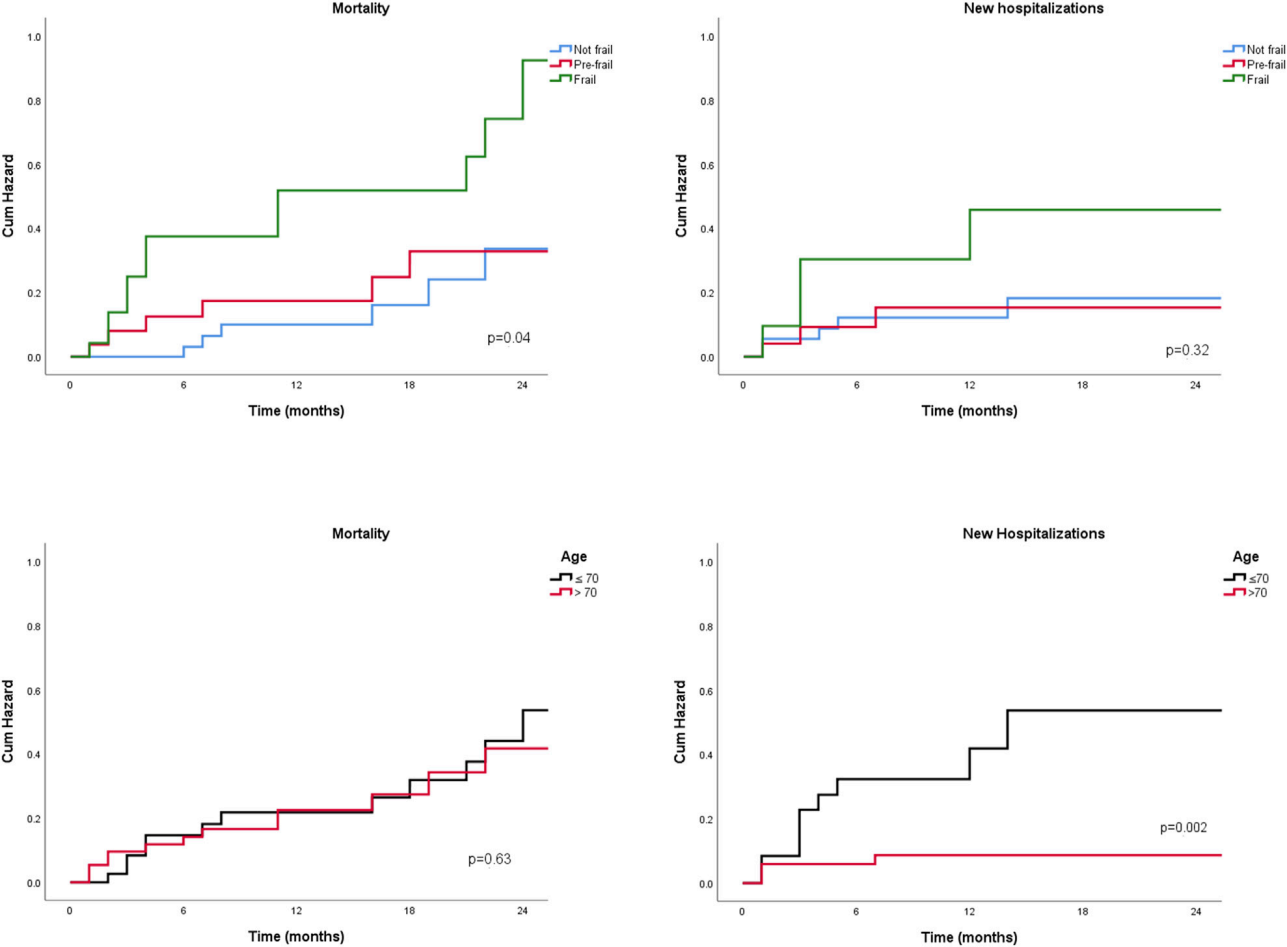
Two-years probability for new hospitalization and mortality, by age groups and by frailty degree.

Clinical outcomes were analyzed across the two age groups, focusing on the onset of disease decompensation symptoms, hospitalizations, listing and delisting, and transplant-free survival. No significant differences were observed in the prevalence of clinical complications—including spontaneous bacterial peritonitis, hepatic encephalopathy, hepatorenal syndrome, or acute-on-chronic liver failure—between older and younger patients (data not shown). Younger patients had a higher probability of being listed for liver transplantation (57.5% vs 35.0%, p = 0.04) and were more likely to undergo transplantation (69.5% vs 38.0%, p = 0.001). However, their hospital stays were significantly longer than those of older patients [21 (14–40) days vs 12 (7–15) days, p = 0.02; data not shown]. Younger patients also had a higher number of hospitalizations during follow-up (p = 0.03) and a greater probability of hospitalization at 24 months (p = 0.002) (Figure 1). No significant differences were found in transplant-free survival between the two groups (Figure 1). Regarding frailty assessment, the incidence of clinical complications did not significantly differ among frailty groups. Similarly, the proportion of patients listed for liver transplantation was comparable across groups (data not shown). However, the rate of actual transplantation varied: 41.4% of robust patients, 50.0% of pre-frail patients, and 77.8% of frail patients underwent transplantation, though this difference was not statistically significant. Hospitalization rates were significantly higher among frail patients, with 48.0% of frail individuals requiring admission, compared to 17.0% of robust patients and 28.5% of pre-frail patients. However, the overall probability of hospitalization did not reach statistical significance (Figure 1). Survival rates differed significantly based on frailty status, with survival probabilities of 80.4% for robust patients, 81.5% for pre-frail patients, and 52.0% for frail patients (p = 0.026). The probability of death also varied significantly among frailty groups (p = 0.04) (Figure 1). When stratifying patients by MMSE median values, frailty was associated with a significantly higher probability of mortality (p = 0.04) and new hospitalizations (p = 0.03) among those with lower MMSE scores. In this low-MMSE subgroup, older patients also had a significantly higher likelihood of hospitalization (p < 0.001; data not shown). Additionally, in a sensitivity analysis conducted exclusively on patients with HCC, frail individuals exhibited a significantly higher probability of death (p = 0.008), while older patients demonstrated an increased likelihood of hospitalization (p = 0.007; data not shown).

### Predictors of overall survival

**FIGURE 2:**
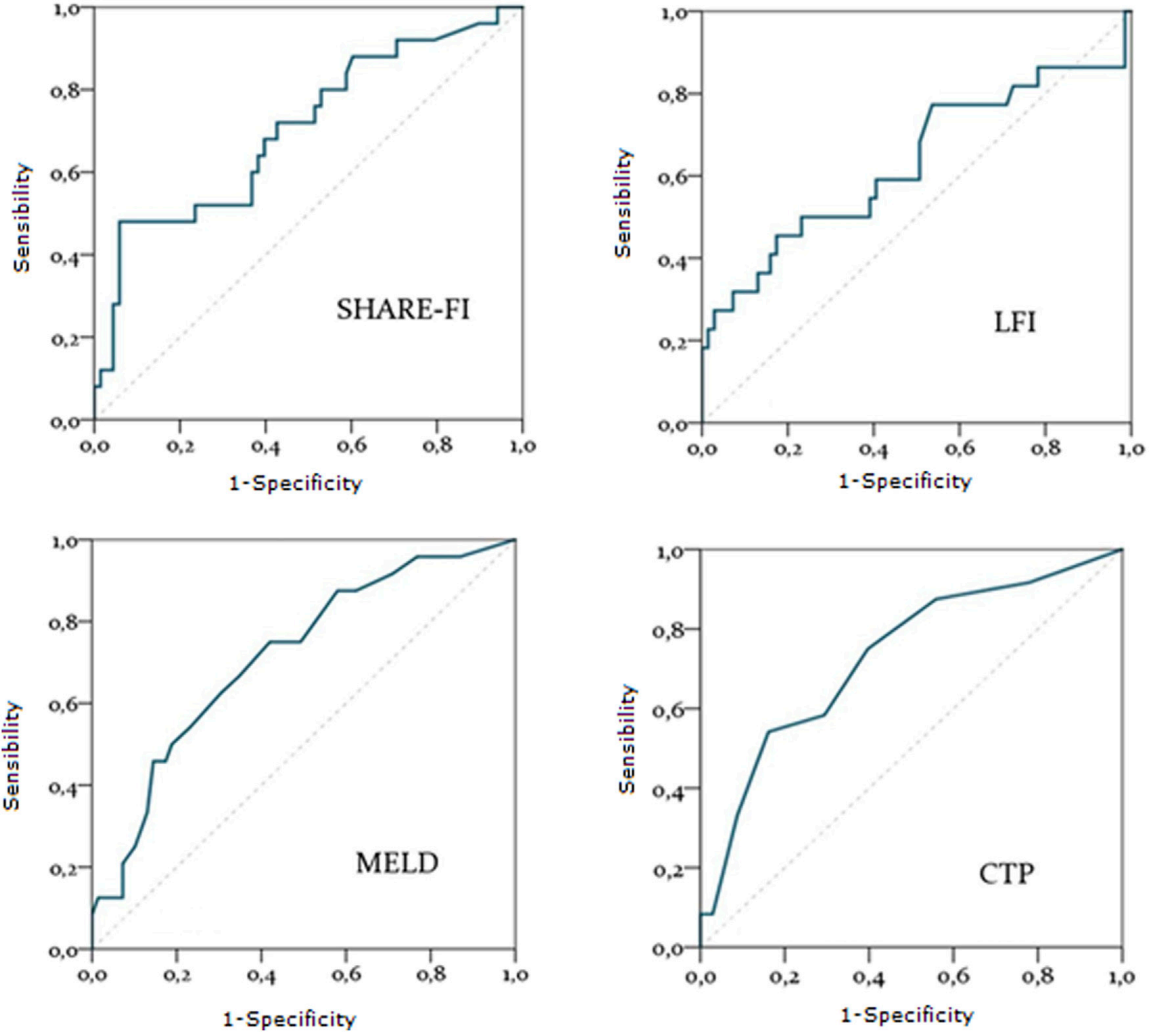
Diagnostic accuracy of SHARE-FI, LFI, MELD and CTP in predicting mortality. Abbreviations: SHARE-FI, Frailty Instrument of the Survey of Health, Ageing and Retirement in Europe; LFI, Liver frailty Index; MELD, Model for End stage Liver Disease; CTP, Child-Turcotte-Pugh score.

A total of 24 patients underwent liver transplantation. During the observation period, 26 patients died, including six post-transplant deaths. The predictive accuracy of variables such as MELD and CTP scores for transplant listing, as well as frailty scores (SHARE-FI and LFI), was assessed using ROC curves. MELD and CTP scores demonstrated strong predictive accuracy for mortality risk [AUC = 0.71 (0.60–0.83), p = 0.002 and AUC = 0.73 (0.61–0.85), p = 0.001, respectively]. Among frailty scores, only SHARE-FI showed significant predictive power [AUC = 0.71 (0.58–0.83), p = 0.003], whereas LFI did not reach statistical significance [AUC = 0.63 (0.48–0.78), p = 0.21] (Figure 2).

The multivariate Cox models for mortality prediction (Table 2) highlight the associations between clinical and frailty indicators and overall mortality risk, including among liver transplant recipients. MELD remained significantly associated with mortality across all models [HR = 1.17, 95% CI: 1.07–1.29, p = 0.001 and HR = 1.11, 95% CI: 1.02–1.21, p = 0.002]. Among frailty indicators, only LFI demonstrated a significant association with mortality (HR = 1.69, 95% CI: 1.03–2.77, p = 0.04). Similarly, CTP was significantly associated with mortality in both models in which it was included [HR = 1.43, 95% CI: 1.35–1.81, p = 0.003 and HR = 1.41, 95% CI: 1.10–1.79, p = 0.006]. Conversely, neither SHARE-FI nor LFI showed significant associations in other models [HR = 1.08, 95% CI: 0.83–1.40, p = 0.60 and HR = 1.30, 95% CI: 0.75–2.26, p = 0.35, respectively]. In the sensitivity analysis conducted on patients with HCC (Table 3), MELD remained the only significant predictor of mortality [HR = 1.18, 95% CI: 1.02–1.36, p = 0.03], while no significant associations were observed for frailty indicators or CTP. All models retained statistical significance even after adjusting for MMSE (data not shown).

**TABLE 2:** Multivariate cox models for mortality prediction.

Variables	Model 1	Model 2	Model 3	Model 4	Model 5
	HR (CI95%), p-value	HR (CI95%), p-value	HR (CI95%), p-value	HR (CI95%), p-value	HR (CI95%), p-value
AGE	0.97 (0.93; 1.01), p = 0.18	0.95 (0.91; 1.03), p = 0.19	0.97 (0.93; 1.02), p = 0.16	0.97 (0.93; 1.03), p = 0.24	0.97 (0.93; 1.01), p = 0.26
MELD	1.62 (1.08; 1.25), p < 0.001	1.17 (1.07; 1.29), p = 0.001	1.11 (1.02; 1.21), p = 0.002	—	—
CTP	1.49 (1.21; 1.85), p < 0.001	—	—	1.43 (1.35; 1.81), p = 0.003	1.41 (1.10; 1.79), p = 0.006
LFI	1.91 (1.18; 3.11), p = 0.009	—	1.69 (1.03; 2.77), p = 0.04	—	1.30 (0.75; 2.26), p = 0.35
SHARE-FI	1.28 (1.03; 1.60), p = 0.003	1.07 (0.83; 1.39), p = 0.59	—	1.08 (0.83; 1.40), p = 0.60	—

Notes: Model 1 was adjusted for sex, age, and liver disease etiology. Model 2 included these factors along with MELD, and SHARE-FI, while Model 3 was adjusted for MELD, and LFI., Model 4 incorporated sex, age, liver disease etiology; CTP, and SHARE-FI, and finally, Model 5 included CTP, and LFI, as additional predictors.

Abbreviations: MELD, model for end stage liver disease; CTP, Child-Turcotte-Pugh score; MPI, multidimensional prognostic index; MMSE, mini mental state examination; SHARE-FI, frailty instrument of the survey of health, Ageing and Retirement in Europe; LFI, liver frailty index.

**TABLE 3:** Multivariate Cox Models for Mortality Prediction in patients with HCC.

Variables	Model 1	Model 2	Model 3	Model 4	Model 5
	HR (CI 95%), p-value	HR (CI 95%), p-value	HR (CI 95%), p-value	HR (CI 95%), p-value	HR (CI 95%), p-value
AGE	0.96 (0.88; 1.05), p = 0.37	0.98 (0.88; 1.09), p = 0.65	0.93 (0.83; 1.04), p = 0.20	1.02 (0.89; 1.16), p = 0.81	0.97 (0.85; 1.10), p = 0.61
MELD	1.22 (1.08; 1.37), p = 0.001	1.18 (1.02; 1.36), p = 0.03	1.09 (0.90; 1.31), p = 0.37	—	—
CTP	1.52 (1.07; 2.16), p = 0.02	—	—	1.50 (0.83; 2.38), p = 0.14	1.31 (0.85; 2.04), p = 0.22
LFI	2.92 (1.16; 7.35), p = 0.02	—	2.01 (0.58; 7.02), p = 0.27	—	1.97 (0.55; 7.02), p = 0.30
SHARE-FI	1.52 (1.01; 2.29), p = 0.04	1.34 (0.81; 2.21), p = 0.25	—	1.37 (0.91; 2.05), p = 0.13	—

Notes: Model 1 was adjusted for sex, age, and liver disease etiology. Model 2 included these factors along with MELD, and SHARE-FI, while Model 3 was adjusted for MELD, and LFI., Model 4 incorporated sex, age, liver disease etiology; CTP, and SHARE-FI, and finally, Model 5 included CTP, and LFI, as additional predictors.

Abbreviations: MELD, model for end stage liver disease; CTP, Child-Turcotte-Pugh score; MPI, multidimensional prognostic index; MMSE, mini mental state examination; SHARE-FI, frailty instrument of the survey of health, Ageing and Retirement in Europe; LFI, liver frailty index.

## Discussion

Our study highlights frailty and its severity as key predictors of post-transplant survival in older cirrhotic patients, whereas chronological age alone was not associated with mortality in this population.

In recent years, advancements in antiviral therapies and treatments for decompensated liver disease, alongside improvements in surgical techniques, have significantly reshaped the epidemiology of liver disease. Consequently, the proportion of cirrhotic patients within the geriatric population has risen, prompting critical discussions in hepatology, particularly regarding liver transplantation (Su et al., 2016). The increasing number of transplant-eligible patients, coupled with the rising age of recipients, underscores the need to refine criteria for both patient selection and organ allocation (Su et al., 2016).

Our study focused on frailty, a geriatric syndrome associated with increased vulnerability to stressors (Terziotti et al., 2023), examining its role both as a potential criterion for liver transplant selection and as a predictor of mortality. While interest in the impact of frailty in surgical and transplant settings has grown in recent years, our study stands out as one of the most relevant in this field. Our findings highlight that preoperative assessment should not rely solely on chronological age but should instead incorporate a thorough frailty evaluation, which appears to be a key determinant of post-transplant outcomes. Our data confirm that frail patients had significantly lower survival rates and a higher risk of mortality. These findings align with existing geriatric literature, which consistently demonstrates that frailty is associated with worse mortality outcomes, regardless of the assessment tool used (Ekram et al., 2021), with effects extending up to 10–18 years (Salminen et al., 2020). Within this framework, frailty is undoubtedly influenced by factors such as sarcopenia, a well-known risk factor that, in a vicious cycle, further exacerbates its progression and associated adverse outcomes. In the context of cirrhosis, sarcopenia has been linked to reduced survival, although some degree of recovery after liver transplantation has been reported. Notably, it affects approximately 40% of patients on the liver transplant waiting list and has been shown to double the risk of death while awaiting transplantation. Importantly, this increased mortality risk has been found to be independent of MELD scores, suggesting that sarcopenia could serve as a crucial determinant of frailty and a key prognostic factor in this patient population (Soldera, 2018). Moreover, the risk of mortality in frail patients was higher than in pre-frail individuals. Notably, clinical outcomes were worse in frail patients with lower MMSE scores. This finding is not surprising, given that the prevalence and severity of frailty increase as MMSE scores decline. Supporting this evidence, a study conducted on outpatients attending CCDDs centers in Lombardy, Italy, reported that the prevalence of severe frailty ranged from 7.2% among individuals with an MMSE score of 24 or higher to 24.2% among those with an MMSE score below 10 (Bellelli et al., 2023). These findings suggest that implementing structured interventions aimed at improving physical and nutritional status before transplantation could be beneficial in optimizing clinical outcomes. A particularly intriguing aspect of our study is the lack of significant differences in transplant rates among frail, pre-frail, and robust patients. Although frailty is recognized as a negative prognostic factor, no official criteria currently exclude frail patients from transplant eligibility. Instead, transplant selection teams primarily consider disease severity, organ functional reserve, and comorbidities rather than frailty alone. It is plausible that frail patients, who exhibited higher MELD scores, were prioritized for transplantation regardless of their frailty status. This could explain the absence of significant differences in transplant rates across the groups.

Regarding predictors of mortality in liver transplantation, both the MELD score and the CTP score have demonstrated strong prognostic value (Ekram et al., 2021). The MELD score is a crucial tool for assessing mortality risk in patients awaiting transplantation, with higher scores directly correlating with increased mortality. Patients with elevated MELD scores face greater risks of death both while on the waitlist and post-transplant (Kim et al., 2023). Similarly, the CTP score has been linked to post-transplant mortality, with patients scoring below 10 exhibiting significantly better survival rates than those with scores of 10 or higher (Yao et al., 2004). This finding aligns with our ROC curve analysis and regression models for estimating 24-month mortality risk across the entire sample, in which both MELD and CTP consistently emerged as significant predictors of survival. In our analysis of frailty as a potential predictor of mortality in this population, we found that frailty was a significant determinant of overall mortality, but only when assessed using the LFI. This finding may seem paradoxical, given that our ROC curve analysis indicated that the SHARE-FI demonstrated promising predictive ability, whereas the LFI did not. The LFI and SHARE-FI are among the most widely used tools for assessing frailty in liver transplant recipients (Haugen et al., 2020; Salminen et al., 2020; Soldera, 2018; Kim et al., 2023). Notably, the LFI, developed by Lai et al., in 2019, has been validated as a reliable predictor of mortality risk in cirrhotic patients on the transplant waiting list (Lai et al., 2019). The discrepancy observed in our study may stem from fundamental differences between these two indices. The LFI incorporates factors particularly relevant to mortality risk, such as functional limitations and malnutrition risk, which directly impact survival and are specifically tailored to patients with liver disease. It aligns with Linda Fried’s frailty criteria, which define frailty as the presence of at least three of the following: involuntary weight loss, reduced muscle strength (handgrip), slow walking speed, low levels of physical activity, and increased fatigability (Fried et al., 2001). In contrast, SHARE-FI focuses on broader health status indicators that may not correlate as strongly with mortality risk in this specific patient population (Romero-Ortuno et al., 2011). Our findings suggest that the LFI may be the most clinically useful index for estimating mortality risk in older patients undergoing liver transplantation. In our analysis, the LFI and SHARE-FI showed a significant association with mortality in certain models, suggesting an independent prognostic value. However, when included in more comprehensive models (e.g., Model 5 for LFI and Model 2 for SHARE-FI), this effect was no longer significant, which may indicate that frailty shares part of its predictive capacity with liver disease severity. These findings suggest that frailty may not only be an independent risk factor but also a marker of clinical deterioration in patients with advanced cirrhosis. However, its specific contribution relative to traditional disease severity scores warrants further investigation in larger cohorts with longitudinal assessments. Among patients with HCC, only MELD remained a significant predictor of mortality, while no meaningful associations were observed between CTP, frailty indicators, and mortality. Notably, patients with HCC in our cohort were older than those without HCC but were also more frequently classified as robust. This may partly explain why frailty did not emerge as a predictor of mortality in this subgroup. In contrast, traditional liver function metrics may provide more reliable mortality predictions in this population. The MELD score specifically evaluates liver function and its associated complications, offering a direct assessment of the physiological factors influencing mortality risk. Frailty, on the other hand, does not necessarily correlate with liver function severity, meaning that patients with high frailty scores may still exhibit relatively preserved liver function.

Previous studies have largely focused on the role of age in geriatric patients undergoing liver transplantation. Although recent updates to transplant guidelines no longer consider age a strict limiting factor (Huang et al., 2023; Su et al., 2016), older patients are often burdened with increased comorbidities and functional decline, which can render them ineligible for transplantation. For instance, Leibovici-Weissman et al. identified age as a key predictor of long-term post-transplant survival, independent of MELD score (Leibovici-Weissman et al., 2017). Similarly, Sharpton et al. demonstrated that both high MELD scores (≥28) and advanced age (≥70 years) independently increase the risk of graft loss, with a synergistic effect that significantly worsens outcomes when both factors are present (Sharpton et al., 2014). In our study, age did not significantly influence mortality among older cirrhotic patients. This finding underscores two important considerations. First, age alone may not fully account for the impact of other critical factors, such as comorbidities, which can strongly affect patient prognosis (Radonjić et al., 2022). Second, assessing mortality risk in liver disease is inherently complex, requiring a multifactorial approach. Interestingly, younger patients exhibited a higher probability of new hospitalizations. This may, in part, be attributed to the fact that, in our sample, younger patients had a higher prevalence of alcohol-related liver disease, which is known to be associated with a more aggressive disease course, higher rates of decompensation, and increased healthcare utilization (Matovic Zaric et al., 2024). Alcohol-related liver disease often presents with acute exacerbations, including episodes of alcoholic hepatitis, infections, and gastrointestinal bleeding, which frequently require hospitalization (Osna et al., 2024). Furthermore, clinically, younger individuals are more likely to present with acute conditions that necessitate frequent hospital visits for management and stabilization. In contrast, older patients often follow a more chronic disease trajectory, managing their established comorbidities more effectively and consequently requiring fewer hospitalizations (Mohan et al., 2022). Healthcare access and utilization patterns may also contribute to this trend. Younger patients might be more inclined to seek emergency care rather than engage in regular follow-ups, potentially due to a lack of awareness regarding the importance of ongoing disease management. Additionally, barriers such as insurance coverage and limited access to specialized care can delay necessary treatments, exacerbating conditions and leading to increased hospitalizations. Although not explicitly assessed in our study, psychosocial factors likely play a significant role in these differences. Younger individuals often struggle with adherence to medical regimens, a key component in managing chronic liver disease. Poor adherence can lead to worsening symptoms and an increased need for hospitalization (Leibovici-Weissman et al., 2017; Sharpton et al., 2014; Radonjić et al., 2022). Frailty, however, did not appear to influence hospitalization rates, except in patients with low MMSE scores, where frailty was associated with a higher probability of readmission within 24 months. Cognitive impairment may interact with frailty in a way that amplifies health risks. It can impair a patient’s ability to manage their condition, adhere to treatments, and recognize early signs of deterioration, ultimately increasing the likelihood of hospitalization and readmission (Rezaei-Shahsavarloo et al., 2020).

Our findings prompt a broader reflection on the concept of frailty in the context of liver transplantation. While traditionally considered an independent prognostic factor, frailty may also serve as a marker of disease severity, reflecting the cumulative impact of end-stage liver disease (ESLD) progression rather than being solely an age-related condition. This raises the question of whether frailty should be regarded not only as a modifiable risk factor but also as a marker of disease severity itself. From this perspective, frailty could reflect the cumulative burden of systemic inflammation, sarcopenia, and multi-organ dysfunction that accompany ESLD progression, rather than being merely an age-related syndrome. Recognizing frailty as an intrinsic component of ESLD progression rather than an isolated variable could have important implications for patient management. Instead of viewing frailty purely as a separate entity requiring independent intervention, a more comprehensive approach might involve addressing both frailty and liver disease severity simultaneously. This could entail optimizing nutritional and physical rehabilitation strategies alongside standard hepatologic care to mitigate the impact of frailty on clinical outcomes. Future studies should further investigate the dynamic interplay between frailty and disease progression, exploring whether targeted interventions aimed at improving frailty could also influence the natural history of ESLD and transplant outcomes. These considerations highlight the need for future longitudinal research to better delineate the interplay between frailty and liver disease progression.

### Limitations and strengths

Several limitations of this study should be acknowledged, particularly the small sample size, which restricts the generalizability of our findings. However, the study’s distinct focus on geriatric patients makes it one of the few to specifically address this critical population in the context of liver transplantation. Additionally, further investigation into the interplay between cognitive function and frailty in this cohort would be valuable, as a deeper understanding of this relationship could offer important insights into the multifaceted nature of frailty in older liver transplant candidates.

## Conclusion

Our study highlights that frailty—rather than age—may serve as a reliable predictor of overall mortality in liver transplant recipients. Future research is needed to validate these findings and further refine the assessment of frailty, with the goal of optimizing patient selection for transplantation.

## Key summary points

**Aim:** This study aims to evaluate the impact of age and frailty on clinical outcomes, including hospitalizations, waitlist survival, and post-transplant mortality, in cirrhotic older patients undergoing evaluation for liver transplantation.

**Findings:** Younger patients had a higher risk of hospitalization during follow-up (p = 0.03) and a greater 24-month hospitalization probability (p = 0.002). MELD, CTP, and LFI scores were significant predictors of mortality, with SHARE-FI showing additional predictive power among frailty indices.

**Message:** Frailty, rather than age, appears predictive of mortality in older liver transplant candidates.

## Data Availability

The raw data supporting the conclusions of this article will be made available by the authors, without undue reservation.
