# Supplementary material for: The impact of age and frailty on hospitalization and survival in older liver transplant recipients: a longitudinal cohort study
Source: Front Aging. 2025 Apr 28;6:1539688. doi: 10.3389/fragi.2025.1539688 (PMC12066423; doi:10.3389/fragi.2025.1539688)
Supplement: Supplementary file 1 [file Table1.docx]

**Supplementary Table 1. Characteristics of the sample according to the presence of frailty.**

| **Variable** | **Robust (n=47)** | **Pre-frail (n=28)** | **Frail (n=25)** | **p-value** |
| --- | --- | --- | --- | --- |
| **Age** | 72.0 (69.3;73.0) | 71.0 (60.3;72.0) | 67.0 (56.5;71.0) | **0.01** |
| **Male sex** | 34 (72.3%) | 18 (64.2%) | 15 (60.0%) | 0.23 |
| **Weight (kg)** | 76.0±13.2 | 73.7±12.8 | 80.7±18.2 | 0.19 |
| **BMI (kg/m^2^)** | 24.2±3.8 | 22.0±4.1 | 33.0±4.7 | 0.21 |
| **Etiology** |  |  |  |  |
| HCV | 9 (20.3%) | 7 (26.9%) | 7 (22.5%) | 0.33 |
| HBV | 10 (23.2%) | 0 | 4 (12.9%) | **0.03** |
| Alcool | 12 (27.9%) | 8 (30.7%) | 11 (35.4%) | 0.45 |
| NASH | 8 (18.7%) | 2 (7.6%) | 5 (20.8%) | 0.34 |
| Altro | 4 (9.3%) | 9 (34.6%) | 4 (12.9%) | 0.27 |
| **HCC** | 35 (76.1%) | 14 (51.9%) | 5 (20.0%) | **<0.001** |
| **MELD at baseline** | 12 (8-15) | 14 (10-19) | 17 (12-23) | **0.003** |
| **CTP at baseline** | 6 (5-8) | 8 (7-11) | 9 (7-11) | **<0.001** |
| **Hospitalizations in the preceding 3 months** | 20 (42.5%) | 7 (25.0%) | 14 (56.0%) | **0.05** |
| **Ascites** | 14 (29.9%) | 14 (50.0%) | 18 (72.0%) | **0.002** |
| **Esophageal varices** | 23 (48.9%) | 14 (50.0%) | 18 (72.0%) | **0.034** |
| **Hepatic encephalopathy** | 5 (10.6%) | 8 (28.5%) | 11 (44.0%) | **0.026** |
| **Multidimensional evaluation** |  |  |  |  |
| CIRS-CI | 2 (2;4) | 3 (2;4) | 2 (2;5) | 0.23 |
| ADL | 6 (6;6) | 6 (5;6) | 5 (5;6) | **<0.001** |
| IADL | 8 (7;8) | 7 (6;7) | 6 (4;7) | **<0.001** |
| MNA | 25.0  (22.4;26.5) | 22.5  (20.4;24.6) | 21.0  (19.0;  22.4) | **<0.001** |
| MMSE | 26.2±3.2 | 25.6±3.2 | 24.1±4.8 | **<0.001** |

*Notes*: numbers are expressed as number (percentages), median (IQR) or mean±standard deviation. *Abbreviations* HCV, Hepatitis C Virus; HBV, Hepatitis B Virus; NASH, Non-Alcoholic SteatoHepatitis; HCC, hepatocellular carcinoma; MELD, Model for End stage Liver Disease; CTP, Child-Turcotte-Pugh score; BMI, body mass index; ADL, Activity of Daily Living; IADL, Instrumental Activity of Daily Living; MNA, Mini Nutritional assessment; MMSE, Mini Mental State Examination; MPI, Multidimensional Prognostic Index.
